# Maternal mortality study in the Eastern Democratic Republic of the Congo

**DOI:** 10.1186/s12884-022-04783-z

**Published:** 2022-05-31

**Authors:** Imani Bin-Eradi Ramazani, Simon-Decap Mabakutuvangilanga Ntela, Mathieu Ahouah, Daniel Katuashi Ishoso, Rothan-Tondeur Monique

**Affiliations:** 1 Nursing Sciences Research chair, Laboratory Educations and Health Practices (LEPS), Université Sorbonne Paris Nord, (EA 3412), (UFR SMBH), F-93017 Bobigny, France; 2 Nursing Sciences Section, Institut Supérieur des Techniques Médicales de Kindu (ISTM-KINDU), PB.304 Kindu, Maniema Kindu, Democratic Republic of the Congo; 3 Section of Nursing Sciences, Institut Supérieur des Techniques Médicales de Kinshasa, BP 774 Lemba, Kinshasa, Democratic Republic of the Congo; 4 Center for Research in Nursing Sciences and Health Innovation (CReSIIS), Kinshasa, Democratic Republic of the Congo; 5 Department of Community Health, School of Public Health of Kinshasa, University of Kinshasa (UNIKI), B.P. 11850 Kin I, Kinshasa, Democratic Republic of the Congo; 6 Nursing Sciences Research Chair Paris, Assistance Publique Hôpitaux de Paris (AP HP), Paris, France

**Keywords:** Maternal mortality, Performance-based financing, Delay, Democratic republic of the congo

## Abstract

**Background:**

The reduction of maternal mortality in developing countries such as the Democratic Republic of Congo (DRC) still raises many questions. Indeed, this large country in the heart of Africa ranks 4th among the eight countries that alone account for more than 50% of maternal deaths in the world, behind India, Nigeria and Pakistan. However, there is no up-to-date data on maternal mortality in eastern DRC. This study measures the mortality rate rate in health facilities in eastern DRC and identifies the associated risk factors.

**Methods:**

This analytical epidemiological study was based on retrospective data materna deaths recorded in 59 health facilities, in three health zones in the southern part of Maniema province in east DRC. The study was conducted from July 1, 2015 to June 30, 2020. Descriptive, bi and multivariate analyses were used.

**Results:**

The maternal mortality rate was estimated at 620 deaths per 100,000 live births, of which 46% of maternal deaths were related to a parturients’ delayed decision in seeking healthcare in time (first delay). Maternal deaths were significantly associated with extreme ages (≤ 19 years and ≥ 40 years: *p* =  < 0.001), patient parity (in primigravidas and in large multiparas: *p* = 0.001), complications such as hemorrhagic, (*p* =  < 0.001), uterine ruptures:(*p* =  < 0.001), infections, (*p* =  < 0.001), and dystocia (*p* =  < 0.001).

**Conclusion:**

Despite the efforts made by the DRC and its partners in the fight against maternal mortality, women continue to lose their lives when they decide to give birth. The results imply that it is imperative to strengthen both women and health professionals’ knowledge about pregnancy and maternal health and their power to reduce instances of first delay by supporting women in formulating their birth plans.

## Introduction

A reduction in maternal mortality in the Democratic Republic of the Congo (DRC) and several other developing countries is necessary. The DRC is ranked fourth, after India, Nigeria, and Pakistan, among the eight countries that account for more than 50% of maternal deaths worldwide [1]. In 2017, 94% of the 295,000 women who died during or after pregnancy or childbirth were from the developing countries [2]. To reduce maternal mortality, Rollet [3] has argued that women should receive quality healthcare that is universal and accessible and has appropriate technical facilities and sufficient, competent human resources along with a well-organized health system. All these factors improve the quality of care and potentially reduce avoidable maternal mortality and morbidity [4].

With 846 deaths per 100,000 live births [5], the maternal mortality rate in the DRC remains very high despite the measures implemented to reduce it. Causes of death include insufficient follow-up during pregnancy or after delivery and poor quality of care [6]. Currently, data from the second demographic and health survey organized by the DRC (EDS-RDC II 2013–2014) published in September 2014 [7] are being referenced to, which have neither been updated nor are they specific to the DRC context.

Accordingly, the DRC has adopted the performance-based financing (PBF) approach to promote quality maternal health care without financial barriers [8]. Specifically, the DRC provides Universal Health Coverage [9] through the provision of the quality and quantity of care in health facilities[1, 2]. Notably, the PBF approach has been proven in various African countries in the field of pediatric immunization [1, 10–14].

Four years after the PBF approach was implemented in the framework of the Health System Development Project, the maternal mortality rate remained high despite progress. For example, annual reports from Maniema province show a steady decline in maternal deaths, that is, 294 deaths per 100,000 live births in 2017, 170 deaths per 100,000 in 2018, and 282 deaths per 100,000 in 2019 [15]; however, this decrease has not fulfilled the policy objectives. This is why the DRC has publicly declared that reducing maternal mortality is still its main health concern [16].

Moreover, few studies have examined the maternal mortality rate and the risk factors associated with it in health facilities in eastern DRC [55]. Accordingly, this study measures the maternal mortality rate among pregnant women in health facilities in eastern DRC over a retrospective period of five years.

## Method

### Design

This is an epidemiological study that employs retrospective data from July 2015 to June 2020 to estimate the maternal mortality rate. The aims of this study are descriptive and analytical.

### Setting

This study was conducted in southern Maniema province, eastern DRC, in the health zones of Kasongo, Kunda, and Kibombo. These three zones have 59 health structures, the majority of which are in the public sector; 42 health centers (CS); 12 reference health centers (CSR); 2 private sector maternity units; and 3 general reference hospitals (HGR).

Maniema, one of the 26 provinces in the DRC, has an estimated 2,938,101 inhabitants, and women of childbearing age (15–49 years) represent 21% of the total population, which is 617,001 women [17].

### Operational level health facility staff in the DRC

A health zone (ZS) in the Democratic Republic of Congo is a decentralised entity for the planning and development of health activities in the implementation of primary health care; it benefits from the supervision of the intermediate level [17], and operates according to the strategies, standards and directives given by the central level of the health pyramid [56]. The health zone comprises a population of approximately 50,000 to 100,000 inhabitants in rural areas and approximately 100,000 to 250,000 inhabitants in urban areas [56].

A general referral hospital (HGR) is a medical and health facility at the peripheral level of public utility with management autonomy intended to receive patients referred by the health centers [56]. Its mission is to offer services that fall under the complementary package of activities (PCA), but also to support the health centers in developing the quality of care within the framework of the minimum package of activities. Moreover, as a HGR is a health structure at the second level of the peripheral level of the DRC's health pyramid, its capacity is 1 bed per 1,000 inhabitants, i.e. 100 beds for approximately 100,000 inhabitants [56].

A reference health centers (CSR) is an intermediary health structure located between the health centers (CS) and the (HGR). Its mission is to offer both the health care that falls under the minimum package of activities (PMA) and the complementary package of activities (PCA). It also receives patients referred by the health centers [56].

A Health Centre (HC) is a health structure responsible for primary health care activities at the first level of the peripheral health pyramid in the DRC. Its mission is to offer health services that fall under the minimum package of activities (PMA) according to national standards and serves a population of approximately 5,000 to 10,000 inhabitants within a radius of action of 5 to 8 km [56]. It also has the function of serving as a place for deconcentrating the hospital's health care activities to bring services closer to the communities, but also to serve as the population's first contact with the health system [56].

### Population and sampling

The population for this study comprised all pregnant women who attended the targeted health facilities using PBF during the study period. The estimated population of the three health zones was 265,693 in Kasongo, 119,382 in Kibombo, and 331,513 in Kunda; the number of women of childbearing age was 55,796, 25,070, and 69,618, respectively [17]. The choice of these health zones was motivated by their geographical accessibility and stable security situation.

The exclusion criteria included maternal death after 42 days postpartum, maternal accidental or incidental death during gestation, and women who died without having been pregnant [18]. Table 1 presents distribution of causes and their proportion of maternal deaths, While Table 2 definition of some variables.

**Table 1:** Distribution of causes and their proportion of maternal deaths

Delay as a risk factor for maternal death	These factors affect the interval between the onset of an obstetric complication in a pregnant woman and her outcome. These factors delay the parturient’s decision to seek healthcare in time (first delay), delay her arrival at a health facility in time (second delay), and delay the timely provision of adequate healthcare (third delay) [19]
Delay I (R1)	This delay pertains to the decision by the woman, family, or community to go to the hospital for obstetric care when she begins to feel the signs of danger before delivery [20]. It is associated with the sociocultural context, socioeconomic status of the parturient, knowledge of the signs of danger in pregnancy and/or perception of the severity of the disease during pregnancy, previous cost of care, and previous experience with the health system [21]
Delay II (R2)	This delay pertains to the arrival of the parturient at the health facility for obstetric care. It refers to the problems of accessibility—the distance between the woman’s home and the health facility, poor road infrastructure, cost of transportation, and distribution of health facilities in the health zone where she resides—that prevent her from timely arriving at the hospital [22]
Delay III (R3),	This delay pertains to obstetric care of the woman by the healthcare personnel in a health facility. This factor is linked to the service offered by the health facility to the parturient and refers to the incompetence of the healthcare personnel and the insufficiency or absence of materials, medical equipment, supplies (medicines), and qualified personnel who could provide a suitable environment and other essential factors [23]

**Table 2:** Definition of some variables

Variables	Définitions	Types	Modalities		Rationale
Sociodemographic, clinical and therapeutic characteristics of respondents					
Age	This is the age range of respondents between: ≤ 19 and ≥ 35 years Continuous and categorical		≤ 19 20–24 25–29 30–34 ≥ 35		May explain maternal mortality
Educational level,	These are the trainings that the respondent had taken: either primary, secondary or higher or nothing at all	categorical	Unschooled Primary Secondary Superior		May explain maternal mortality
Profession	These are activities that the respondent performs in life	categorical	Unemployed Farmer Pupil Trader Official		May explain maternal mortality
Religion	This is the church in which the respondent prays	categorical	Muslim Catholic Protestant Kimbanguist Church of awakening Other (s) to be specified		May explain maternal mortality
Marital status	This is the marital status of the respondent who lives in union or alone	categorical	Married Single Divorcee Widow		May explain maternal mortality
Parity	These are the number of deliveries that the respondent has already experienced in her life	categorical	Primiparité(1accouch) Paucipares (2 accouch) Multipares (3–5 accouch) Grande multipares (6 accouch et plus)		May explain maternal mortality
Gesture	These are the numbers of pregnancies that the respondent has already had	categorical	Too early (before 19 years old) Too much reproached (before 2 years) Too many (more than 5 deliveries) Too late (over 35)		May explain maternal mortality
Prenatal care (ANC)	These are the numbers of times the respondent attended antenatal care sessions according to the immunization schedule	categorical	ANC 0 ANC 1 ANC 2 ANC 3 ANC 4		May explain maternal mortality
Use of partograph	This concerns the use of the partograph by nursing staff for monitoring the labor and delivery of respondent	dichotomo us	Yes No Not available		May explain maternal mortality
Liability (3 delays)	This is the responsibility for the occurrence of the death of a parturient	categorical	R1 (Delay in decision-making by the parturient or the family, R2. (Late arrival at the structure) R3. (Delay in taking charge)		May explain maternal mortality
Variables relating to the timing of maternal deaths					
Times of death	This is the period during which the death of the respondent woman occurred	categorical	Prepartum Perpartum Postpartum	Important variable	
Variables relating to the causes of death in kindergartens					
Direct causes of maternal mortality	These are the direct medical causes which caused the death of the respondent	categorical	Bleeding, Infections Eclampsia Abortions Obstructed labor Uterine rupture		Important variable
Indirect causes of maternal mortality	These are the indirect medical causes which caused the death of the respondent	categorical	Anemia Malaria Tuberculosis HIV / AIDS Gastrointestinal bleeding Diabetes Pulmonary embolism Cardiovascular patients Kidney failure		Important variable

### Data collection

The method used to collect the information took into account the data collected at two different periods data of five (5) years and those of one (1) year). This approach has already been developed in the study conducted by Reinke and his colleagues. [49]. Indeed, the authors justified this procedure by the fact that the data collected over a long period were considered incomplete or not representative. In this regard, Ntambue explains that if in developed countries the system for registering maternal deaths is considered effective, it seems utopian in developing countries where the national health information system (SNIS) is experiencing real weakness [5].

Thus, the five-year data including 97,000 women were collected in all the health structures under study (health centers, reference health centers, general reference hospitals and maternities), but only allowed the determination of the number of pregnant women who have given birth, their maternal mortality rate, the time of death and the delays justifying these deaths, without giving more details. This in view of the weaknesses linked to poor archiving and poor filling in of information in health centers [50]. This state of affairs was already noted at the seventy-second WHO assembly of the eleventh revision of the international classification of diseases (ICD-11) during which member states recognized that despite the efforts made, the system of notification, registration and declarations of maternal deaths and obstetric causes is still lacking at the community level and in first-level primary health care facilities [18].

In addition, in view of all these shortcomings, a second data collection was deemed necessary, for a period of one year. This was carried out exclusively in referral health structures including general referral hospitals (HGR), referral health centers (CSR) and maternity units. This made it possible to generate complete and specific data, with a great possibility of carrying out more detailed analyzes on 5085 women.

Data were collected electronically using a data entry mask programmed into the KoBoCollect® server and downloaded to tablets. KoBoCollect is an open source software that is used for the collection, management and use of offline data with mobile devices such as, tablets, smartphones, mobile phones even in very remote areas and contexts of limited means. It also allows the construction of a questionnaire through a web interface [52].

Based on this data, a team of six members (one doctor and five nurses) with extensive experience in primary healthcare was selected in October 2020. They were trained for two days by the principal investigator on the data collection tools (KoBoCollect®: installation on the tablets, data recording, and transmission in the pourer), research considerations, collection of information from primary and secondary sources, research on maternal deaths in the community, and cross-referencing data collected in health structures (i.e., CS, CSR, HGR, and maternity units) and those consolidated at the central offices of health zones.

Documentary analysis of the survey questionnaires was used to identify maternal deaths.

The questionnaires were designed using a Microsoft Excel spreadsheet and deployed on the KoBoCollect® platform. The data sources were consultation registers, delivery registers, obstetric records, operative reports of pregnant women, antenatal consultation registers and records, and death registers.

A test with a sample of 40 files (10 for each interviewer and 10 for the monitor) containing the same characteristics as the participants in this study was carried out at Kindu General Hospital. The purpose of this test was threefold: to ensure that the interviewers had mastered the use of the KoBoCollect® tool such that they would achieve the desired objectives, to compare the questionnaire with the information contained in the various information sources for the final validation of the questionnaire, and to assess the participants’ understanding of the questionnaire and estimate its duration.

A test with a sample of 40 files (10 for each interviewer and 10 for the monitor) containing the same characteristics as the participants in this study was carried out at Kindu General Hospital.

To collect the data for this study and include as many subjects as possible, an exhaustive collection was sought in the targeted health facilities during the five-year period; the most detailed analyses were conducted in the last year.

### Data quality control

The following steps were used to control the quality of the data collected:

First, at the data collection level, this study examined the juxtaposition of the data collected from health facilities and the data from the central offices of the health zones by the interviewers, the monitor, and the database manager. This check was used to eliminate duplicates. A cross-check was conducted by the monitor on samples in certain health facilities, and the error rate was 2%. The principal investigator directly contacted some managers of the health facilities for which discrepancies were observed between the data collected by the investigators and the cross-check by the monitor.

### Statistical processing and analysis of data

Statistical analyses were conducted using STATA version 15 software at the significance level α = 0.05. Descriptive statistics comprised presenting the characteristics of the study population. The mean and standard deviation were used as a measure of central tendency and dispersion for the variable “age” as normally distributed and percentages for the categorical variables. The maternal death rate was calculated and presented per 100,000 live births. For the bivariate analyses, the percentages of death in the modalities of the categorical variables were compared using Pearson’s chi-squared test when the numerical conditions for application were fulfilled; otherwise, the exact file test was used. For logistic regression, automatic FORWARD selection was used to identify independent predictors of maternal deaths in health facilities. Thus, only variables significantly associated with maternal deaths after automatic selection were included in the final model. The odds ratio (OR) with its 95% confidence interval (CI) was reported to assess the strength of associations between the variables.

### Ethical considerations

Prior to data collection, the study objectives were explained in detail to the survey participants. The ethical approval of the National Health Ethics Committee of the Ministry of Health of the Democratic Republic of Congo has been obtained. Authorizations were also requested and obtained from the Secretary General of Health of the DRC as well as the head of the provincial health division of Maniema. Free written consents were also obtained from each study participant. Anonymity and confidentiality were guaranteed to all participants in all stages of data manipulation from collection, through processing. They were also initially informed that participation in the survey was voluntary.

## Results

### Description of the study population

This study assessed 97,140 women, who represented 13.5% of the total population of the three chosen health zones.

### Maternal mortality rate

**Fig. 1:**
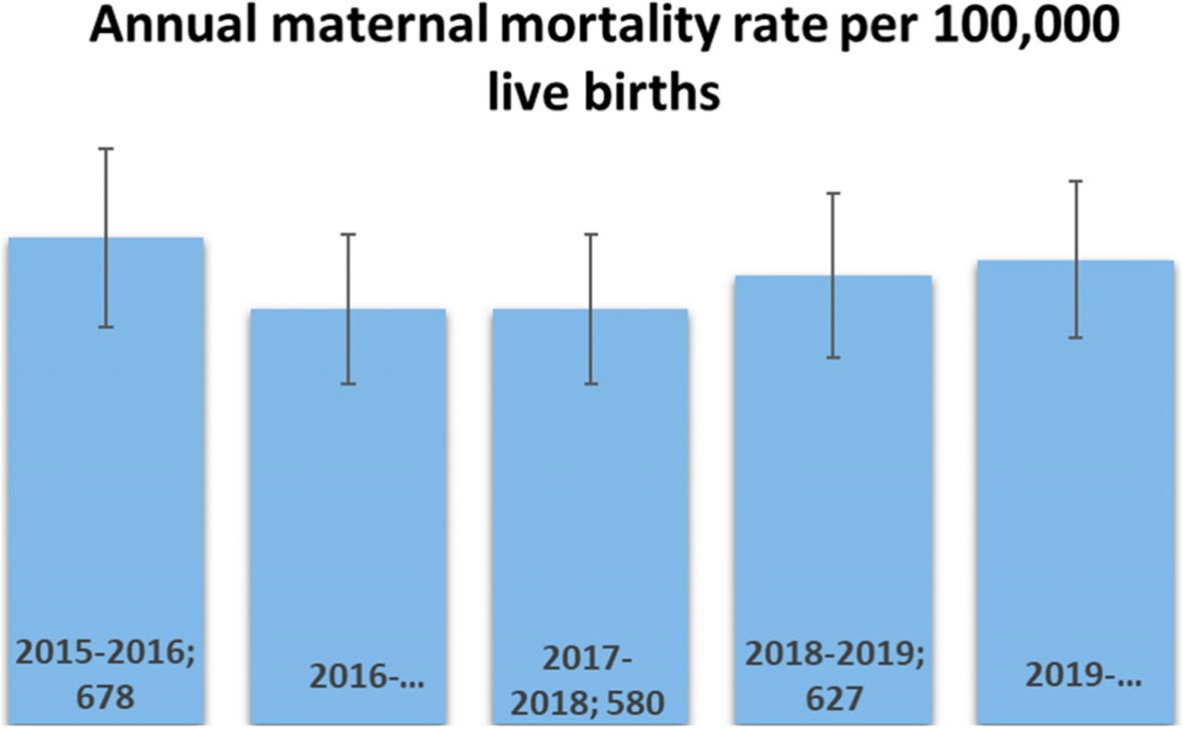
below shows presents the annual maternal mortality rate per 100,000 live births

**Fig. 2:**
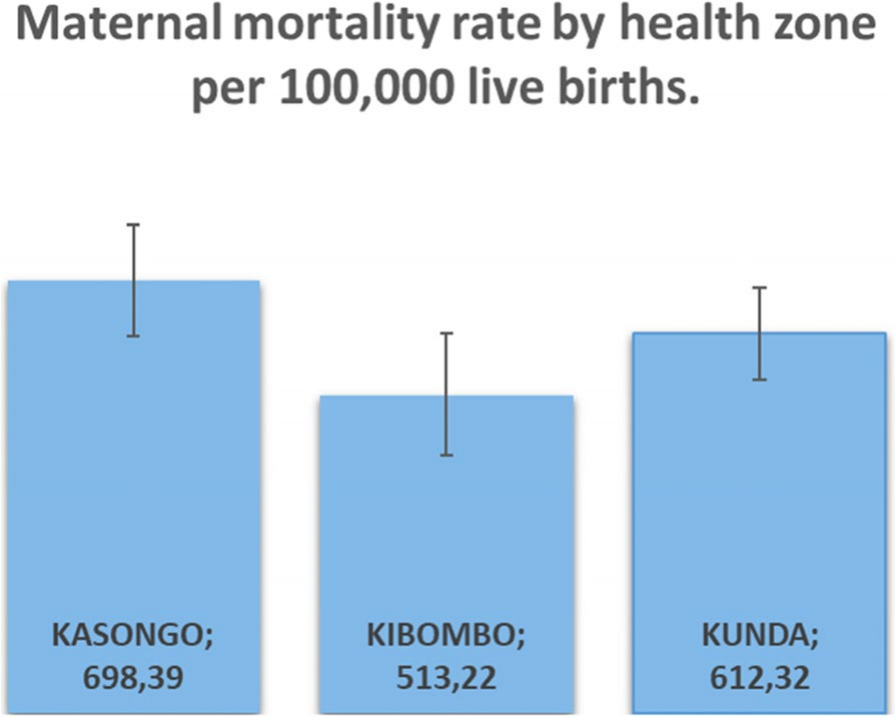
Maternal mortality rate by health zone per 100,000 per 100,000 live births

Of the 97,140 women who gave birth during the observation period, 603 died either in the community or in the CS, CSR, HGR, and maternity units. The corresponding mortality rate was 620 deaths per 100,000 live births. Figure 1. below shows presents the annual maternal mortality rate per 100,000 live births. While Fig. 2. Maternal mortality rate by health zone per 100,000 per 100,000 live births. Furthermore, Table 3 presents the distribution of causes and their proportion of maternal deaths.

**Table 3:** Distribution of causes and their proportion of maternal deaths

Variables	Maternal deaths related to the types of obstetric complications	Number of maternal deaths	Percentage
Direct obstetric causes			
Presence of hemorrhage	326	598	54.5
Uterine rupture	92	600	15.3
Dystocia	87	600	14.5
Infection	68	599	11.3
Abortion complications	55	601	9.2
Pre-eclampsia/eclampsia	18	598	3.0
Indirect obstetric causes			
Anemia	**113**	**600**	**18.8**
Malaria	20	600	3.3
Digestive hemorrhage	10	600	1.7
HIV/AIDS	5	600	0.8
Cardiovascular disease	5	599	0.8
Tuberculosis	2	600	0.3

### Factors associated with the risk of maternal death

In the sample of women, 80% who died were aged 20 years and over, 47% had received primary education, 77% were farmers, 45% were Muslim, and 63% were multiparous and grand multiparous.

Nearly half of the deceased women did not attend the first prenatal consultation, (ANC 1:44%). Among these deceased women, 80% did not complete the recommended fourth antenatal visit Fourth prenatal consultation (ANC 4), and almost half (49.1%) did not use the partogram. Table 4 presents the socio-demographic characteristics of the deceased pregnant women, While Table 5 talks about Clinical and therapeutic characteristics of deceased pregnant women, moreover, Table 6 presents maternal mortality rates by type of delay.

**Table 4:** Socio-demographic characteristics of the deceased pregnant women

Variables	n	%	Med (P25-P75)	Min–Max
Age of pregnant women	600		29 (20–36)	15–45
≤ 19 years	120	20,0		
20 years and over	480	80,0		
Educational attainment of pregnant women	600			
No schooling	115	19,2		
Primary	287	47,8		
Secondary	191	31,8		
Superior	7	1,2		
Occupation of pregnant women	600			
Trader	17	2,8		
Farmer	465	77,5		
Pupil	41	6,8		
Official	16	2,7		
Unemployed	61	10,2		
Religion of pregnant women	601			
Catholic	114	19,0		
Revival Church	120	20,0		
Kimbanguist	23	3,8		
Muslim	271	45,2		
Protestant	73	12,2		
Marital status of pregnant women	599			
Single / divorced	28	4,7		
Married	571	95,3

**Table 5:** Clinical and therapeutic characteristics of deceased pregnant women

Variables	n	%	Med (P25-P75)	Min–Max
Parity	600		4 (1–6)	0–12
Primigest	63	10,5		
Primiparous	98	16,3		
Pauciparous	61	10,2		
Multiparous	216	36,0		
Large multipare	162	27,0		
Carrying out pre-natal consultations (ANC1)	600			
Yes	332	55,3		
No	268	44,7		
Completion of all 4 CPNs (ANC4 completeness)	600			
Yes	117	19,5		
No	483	80,5		
Using the partograph	599			
Yes	305	50,9		
No	294	49,1

**Table 6:** Maternal mortality rates by type of delay

Variables	Maternal deaths related to delays	Total number of maternal deaths delay	Percentage
Delay I (delay in decision making by the woman/family to go to the place of delivery)	275	599	45,9
Delay II (delay in arriving at a health center/hospital)	254	598	45,5
Delay III (delay in getting to the hospital)	225	601	37,4

### Description of the population in 2020

There were 5086 wome Moreover, few studies have examined the maternal mortality rate and the risk factors associated with it in health facilities using PBF in the DRCn representing 5.2% of the total population in three health zones under study. This number comprised all pregnant women who had primarily attended the reference health structures (i.e., HGR, CRS, and maternity units) to give birth.

### Clinical and therapeutic sociodemographic characteristics of parturients

The women included in the study were on average 27 years old and were aged 20–29 years (54%), literate (55.2%), married or in a common-law relationship (96.9%), farmers or shopkeepers (70%), and Muslim (48.8%).

Most women had given birth at least once and had visited a hospital for prenatal consultations (ANC1: 85.2%). A large majority had not completed the recommended four prenatal consultations (ANC4: 18.6%). The partograph has been used effectively in most pregnant women (96.6%). Table 7 presents the sociodemographic characteristics of the respondents. While Table 8 talks about the linical and therapeutic characteristics of the respondents.

**Table 7:** Sociodemographic characteristics of the respondents

Variables	N	%	Moy (± DS)	Min–Max
Age (years)	**5 085**		27 (± 6,8)	14–49
≤ 19 year	792	15,6		
20–29 year	2 774	54,6		
30–39 year	1 194	23,5		
40–49 year	325	6,4		
Educational level	**5 086**			
No schooling	355	7,0		
Primary	1 804	35,5		
Secondary	2 805	55,2		
Superior	122	2,4		
Marital status	**5 085**			
Single	67	1,3		
Divorcee	72	1,4		
Married / common-law	4 928	96,9		
Widow	18	0,4		
Woman's profession	**5 086**			
Unemployed	732	14,4		
Pupil	494	9,7		
Farmer / Trader	3 605	70,9		
Official	255	5,0		
Woman's religion	**5 085**			
Muslim	2 481	48,8		
Catholic	1 090	21,4		
Protestant	863	17,0		
Revival Church	558	11,0		
Kimbanguist	93	1,8

**Table 8:** Clinical and therapeutic characteristics of the respondents

**Variables**	**N**		**%**
Parity	**5 084**		
Primigest	928	18,3	
Primiparous	826	16,3	
Pauciparous	867	17,1	
Multiparous	1 857	36,5	
Large multipare	606	11,9	
Carrying out near-natal consultations (ANC 1)	**5 086**		
Oui	4 333	85,2	
Non	753	14,8	
Completion of all 4 ANC (completion of ANC 4)	**5 077**		
Yes	946	18,6	
No	4 131	81,4	
Using the partograph	**5 081**		
Yes	4 907	96,6	
No	174	3,4	

### Maternal mortality rate in 2020

The number of maternal deaths in the reference health structures (i.e., HGR, CSR, and maternity units) in the last year of the study was 68, which represented 1.3% of the population (i.e., 5018). The mortality rate was 1,300/100,000 live births.

### Determinants of mortality

After adjusting for age group, education, occupation, religion, and marital status of parturients, the risk of maternal death increased significantly for the extreme ages (≤ 19 years and ≥ 40 years; adjusted OR [CI 95%]: 40–49 years = 0.9 [0.4–1.9] and [*p* =  < 0.001]). Regarding clinical characteristics, after adjustment for patient parity, antenatal consultations, and completeness, the risk of maternal death was significantly increased in nulliparous women and in large multiparous women (adjusted OR [CI 95%]: 0.9 [0.5–1.9] and [*p* = 0.001]).

By contrast, in the final logistic regression model of maternal death according to obstetric complications in parturients, after adjustment for independent clinical variables, in-hospital maternal death remained significantly related to complications such as hemorrhage (adjusted OR [CI 95%]: 55.0 [23.6–127.2] and [*p* =  < 0.001]), uterine ruptures (adjusted OR [CI 95%]: 23.1 [5.5–98.1] and [*p* =  < 0.001]), infections (adjusted OR [CI 95%]: 16.5 [4.0–68.0] and [*p *=  < 0.001]), and dystocia (adjusted OR [CI 95%]: 9.7 [3.7–24.9]). Hence, the risk of dying increases in the presence of hemorrhage, uterine rupture, infection, and mechanical dystocia. Table 9 presents Final logistic regression model of maternal death as a function of socio-demographic characteristics, While Table 10 talks about Final model of Logistic regression of maternal death as a function of clinical characteristics. Moreover, Table 11 presents the final model of logistic regression of maternal death as a function of obstetric complications in pregnant women.

**Table 9:** Final logistic regression model of maternal death as a function of socio-demographic characteristics

Variables	Adjusted OR (IC95%)	p
Age groups (years)		** < 0,001**
≤ 19 years	1	
20–29 years	0,3 (0,2 à 0,5)	
30–39 years	0,2 (0,1 à 0,4)	
40–49 years	0,9 (0,4 à 1,9)

**Table 10:** Final model of Logistic regression of maternal death as a function of clinical characteristics

Variables	Adjusted OR (IC95%)	p
Parity		**0,001**
Nulliparous	1	
Primiparous	0,5 (0,3 à 1,1)	
Pauci pare	0,2 (0,1 à 0,5)	
Multiparous	0,3 (0,2 à 0,7)	
Large multipare	0,9 (0,5 à 1,9)

**Table 11:** Final model of logistic regression of maternal death as a function of obstetric complications in pregnant women

Variables	n	% death	Adjusted OR ( IC95%)	p
Haemorrhages	**5 084**			** < 0,001**
Yes	528	8,3	17,2 (10,4 à 28,5)	
No	4556	0,5	1	
Dystocies (DFP…)	**5 082**			0,583
Yes	917	1,5	1,2 (0,7 à 2,1)	
No	4165	1,3	1	
Infections	**5 082**			0,091f
Yes	80	3,8	2,9 (0,9 à 9,6)	
No	5002	1,3	1	
Rupture utérine	**5 080**			**0,038f**
Yes	56	5,4	4,3 (1,3 à 14,2)	
No	5024	1,3	1	
Complications abortives	**5 081**			** < 0,001f**
Yes	12	33,3	39,1 (11,5 à 133,2)	
No	5 069	1,3	1	

## Discussion

This study aimed to measure the mortality rate in health facilities in eastern DRC and identify associated risk factors.

The method used considered data collected in two periods (five years and one year).

The five-year data were collected from all health facilities under study, allowing the determination of the number of pregnant women who gave birth, their maternal mortality rate, the time of occurrence, and delays justifying these deaths. Additional data were unavailable because of limitations linked to health centers’ incomplete files [24]. This state of affairs had been discussed at the Seventy-second session of the World Health Organization for the eleventh revision of the International Classification of Diseases, during which the member states acknowledged that despite the efforts made, the system of notification, registration, and declaration of maternal deaths and obstetrical causes remains limited at the community level and in primary healthcare facilities at the first level [25].

Because of the aforementioned limitations, we conducted a second data collection for one year exclusively from reference health structures, including HGR, CSR, and maternity units. This step made it possible to generate complete, specific data, with a great possibility of conducting all the analyses.

This approach was developed by Reinke and colleagues [26], who justified using this procedure considering that data collected over a long period were considered incomplete or unrepresentative. In this respect, Ntambue states that the maternal death registration system in developed countries considered as efficient seems utopian in developing countries where the national health information system is weak [27].

However, the prominent results of this study reveal that the mortality rate in the health facilities studied (i.e., CS, CSR, HGR, and maternity units) was 620 deaths per 100,000 live births and that the main risk factor was first delay. The results of this study are discussed as follows.

### Maternal mortality ratio

The maternal mortality rate in CS, CSR, HGR, and maternity units was 620 per 100,000 live births.

These results do not fulfill the United Nations Sustainable Development Goals (SDGs), which aims to reduce the global maternal mortality rate to ≤ 70 deaths per 100,000 live births by 2030 and for no country to have a maternal mortality rate more than twice the world’s average [28].

However, considering the World Health Organization principle that no woman should die due to childbirth, we believe that despite the efforts made by the DRC and its partners in the fight against maternal mortality, this rate remains alarming. This observation is supported by a maternal death rate that is three times higher than the rate estimated at the global level in 2017, which was 211 maternal deaths per 100,000 live births [29]. This rate is twice as high as that of developing countries, estimated at 415 maternal deaths per 100,000 live births [30]. By contrast, it is similar to that of sub-Saharan Africa, estimated at 542 deaths per 100,000 live births in 2017 [28].

This maternal mortality rate is 60 times higher than that of Europe, with 10 deaths per 100,000 live births, and 80 times higher than that of Australia, with 7 deaths per 100,000 live births [30], which are industrialized countries.

This high prevalence of maternal mortality rates in our research setting is sufficient justification for the relevance of our study in the Doctoral research program.

### Maternal mortality rate in the reference hospital setting

The mortality rate in reference hospitals (i.e., HGR, CSR, and maternity units) is 1300 maternal deaths per 100,000 live births. Our results are lower than those found by Ntoimo (2,085 per 100,000 live births) [31], Ousmane (1962 per 100,000 live births in the Labé Regional Hospital in Guinea) [32], and Kamga (1,538.9/100,000 live births in three university hospitals in Yaoundé) [33]. However, our results are higher than those found by Sissoko (201.87 per 100,000 live births) [34] and Geleto (149 per 100,000 live births in Ethiopian hospitals) [35]. This diversity of results confirms that maternal mortality in hospitals varies according to the context [36]. Thus, it is difficult to compare the results of industrialized countries with those of African countries or of provinces that have more hospitals with those that have fewer hospitals.

Furthermore, this high rate of maternal death in referral facilities is explained by the fact that most parturients spend more time at first-level facilities and are only referred to a referral facility when obstetric complications appear [31–33].

Regarding our results, our view and that of the aforementioned researchers are the same. First, the referral health structures in our study settings are designated to receive and manage obstetric referrals from the first-level primary healthcare facilities (e.g., dispensary, health post, and health center); second, these facilities confront serious obstetric complications; and third, in the PBF approach, all referrals from first- to second-level health facilities are purchased through a third-party payment supported by the government [9].

### Explanatory factors for in-hospital maternal deaths: Risk markers

The results of this study show that in-hospital maternal death is significantly associated with the age group of the parturient, specifically, the extreme ages (≤ 19 years and ≥ 40 years). After adjustment for independent variables, in-hospital maternal death remained significantly associated with maternal deaths under 20 years of age (*p* < 0.001).

These results corroborate those of Salem and friends [37], who found high maternal mortality in parturients aged ≤ 20 and ≥ 35 years. In this context, the authors explained that in Tunisia, pregnancy outside the age range of 19–34 years is a risk factor for maternal and fetal morbidity and mortality [37]. However, the high mortality observed among adolescents may support the idea of the Europe PMC Funders group that “special attention should be paid to girls under 20 years of age, as they are more at risk than other categories” [38]. Furthermore, our results contradict those of Mouté and Zinvi [39], who suggested that the groups aged 15–19 and 40–44 years were least exposed to maternal deaths, which seems to be confounded by the physiological immaturity and the occurrence of complications in childbirth at extreme ages [39].

In the context of our study, maternal mortality in the group aged ≤ 20 years can be explained, in addition to the aforementioned elements, by sociocultural constraints that force early marriage. Furthermore, the ignorance of young girls could increase the risk of death of parturients aged ≤ 20 years. Thus, this argument sufficiently justifies the interest in good sensitization regarding the prevention of early marriage.

Although the results of our study show that the educational level of the deceased women does not constitute as a factor of mortality in the parturient (p = 0.054), it cannot be eliminated considering the abundant literature on the preponderance of this variable in maternal death at maternity hospitals [40]. On this subject, Baldé explained that a high frequency of of deceased women dying were among those who had low level of education [41]. Similarly, Ousmane study demonstrated that in 69.23% of maternal deaths, the deceased had no education [32]. Based on the aforementioned literature, the low level of education seems to be an important indicator that justifies maternal mortality in this study. In this regard, meh stated that education level is fundamental in explaining the behavior of individuals in a society [40]. Accordingly, low educational level among women leads to low or no use of modern healthcare. Further studies are necessary to confirm the exclusion of this variable in maternal mortality.

Regarding marital status, the results of this study show that this variable is a protective factor for maternal death (*p* > 0.724 ^**f**^). This finding is contrary to that of Kamga, who showed that the majority of parturients who died were single (75%) [33]; according to them, marriage is the ideal setting for sexual activity and procreation; consequently, unmarried women are more likely to die during childbirth. However, because marriage takes place early in this context (as demonstrated in ref. 32 and 42), this sociocultural consideration leads women of childbearing age to enter into an early marriage and have children at a very early age, thus increasing the risk of maternal death.

The results of this study also reveal that the woman’s occupation is not a risk factor in parturient death; however, the differences observed are not statistically significant at the 95% confidence level (*p* > 0.268 f). This result is contrary to that of Ousmane [32] who found that maternal mortality was prevalent among homemakers. However, in our study, attention could be given to women farmers who often conduct heavy fieldwork, thus obliging pregnant farmers to carry large loads that can increase the risk of obstetrical complications.

The results of this study establish no link between the variable religion and the risk of maternal death (*p* ˃ 0.298 f). However, medical literature has demonstrated that religious beliefs significantly influence access to maternal healthcare. For example, certain religious practices and requirements that disrupt social life are harmful [43]. Researchers have argued that religious constraints limit access to maternal healthcare [43]. Thus, this argument supports the hypothesis that religion is a risk factor for death. This idea was supported by Ariyo et al., who demonstrated that Muslim women were 52% more likely to experience maternal deaths (OR: 1.52; CI: 95%: 1.10–2.11) than Christian women [44].

By contrast, in our study, from a demographic view, Muslim religion is predominant in southern Maniema province (Catholics, Protestants, Revivalists, and Kimbaguists were also observed). This cultural diversity also explains the diversity of models of perceptions of the use of modern healthcare, because each religion has a set of traditional health system practices specific to its culture. From a health perspective, the predominance of Muslim women may indicate the lack of social support from Muslim husbands to their wives, which may influence the low utilization of health services. This topic, Al-Mujtaba and al., [53], think in their study on "Assessment of religious influences on the use of maternal health services among Muslim and Christian women", carried out at the center -north of Nigeria, although stated in a Hadith that "A woman should only travel with a Dhu-Mahram (her husband or a man with whom this woman cannot marry at all according to Islamic Jurisprudence), and no man can visit her, except in the presence of a Dhu-Mahram, religion does not seem to see any influence on the choice of attendance of maternal health services. But they believe, on the other hand, that husbands who do not make the necessary arrangements for their wives to attend health services in real time, may contribute to misuse of services [53].

This non-involvement of Muslim husbands would expose parturients to obstetrical complications that could lead to maternal death. On this point, Iliyasu and his colleagues [54], in their study carried out in Ungogo, a community in northern Nigeria, found in their study that only 32.1% of men agreed to accompany their wives for maternity care [54]. Hence, special attention should be given to this issue in our study setting.

The results of this study show that in-hospital maternal death is significantly associated with parity. Thus, the risk of dying increases in primiparous and large multiparous women.

These results corroborate those of Yambare and Yambare and et al. [46] who presented the high risk of maternal death in primiparous and large multiparous women. As in medical literature, the authors justified their results by referring to the physiological immaturity of the deceased primiparous parturients and the occurrence of complications during delivery in large multiparous women [45].

### Causes of maternal death

In relation to the causes of maternal death, in order of importance, the obstetric causes diagnosed on the admission of parturients to the hospital were dystocia (18%), hemorrhage (10.4%), infections (1.8%), and uterine rupture (1.1%). However, after adjustment for independent variables, the obstetrical causes that were significantly associated with maternal death were those found frequently in the literature, mainly in the African region, namely, hemorrhage, uterine rupture, infection, and mechanical dystocia.

Our results are comparable to those of other studies conducted in African countries, especially those conducted by Kamga, Ntoima, Ousmane, Mbeva, and Yambare. Considering their results, these researchers have demonstrated that obstetric complications are either treated late or inappropriately by healthcare personnel [31–33, 46, 47].

In relation to our study environment, our results demonstrated two main types of delayed decision making, namely, that of the parturient or the family to go to the hospital in the event of obstetric complications and that of healthcare personnel at the first level of the health pyramid (health center) to transfer parturients in time in the event of obstetric complications.

### Delays as a major risk factor for maternal deaths

In this regard, Thaddeus and Maine and Actis Danna and colleagues have suggested that women who made decisions in time to go to the place of delivery at the onset of danger signs, women who arrived at the health center or hospital in time to give birth, and women whose hospital admission was adequate are more likely to be saved from obstetric complications than those who experience all three delays [19, 42, 48].

Indeed, the most important risk factor revealed in our study is delay, which could be delay related to the decision by the parturient or the family to go to the place of delivery (first delay), delay related to the arrival at a health center or hospital (second delay), and delay related to hospital management (third delay). Our results show that the cause of death of more than one third of parturients was related to at least one of the three types of delay, and the first delay had the highest proportion (46%).

Our results contradict those of Mbeva, who reported that the highest proportion of maternal deaths was related to the third type of delay (i.e., hospital management; 49%) [47]; they believed that healthcare providers are incompetent in managing obstetrical emergencies in the interaction between members of the healthcare team, management of inputs including blood for transfusion, identification and resolution of obstetrical problems in real time, and use of equipment [47].

Based on of our results, a qualitative study could further our understanding of the reasons for these delays in our study environment.

However, before discussing the results of this study, the method used is discussed.

### Limitations and strengths of this study

The study has some limitations. First, the data were collected in two stages, the first being that of five years where the data were collected in all the health structures (CS, CSR, HGR and maternity). And the second phase is that of a year when the data collections were made in the reference health structures (HGR, CSR and maternities). Second, the data collected during the first step did not allow us to perform specific analyzes that could allow us to associate the dependent variables with our variable of interest. However, the data collected during the second phase, exclusively in the reference health structures (HGR, CSR and maternities), which allowed us to generate complete data and carry out more detailed analyzes, as well as the studied population (nearly 100,000 pregnant women), could justify the power of this study.

## Conclusion

At the end of this study, we can conclude that these results of 620 deaths per 100,000 live births, of which 46% of maternal deaths are linked to the first delay noted in our study, show that women continue to lose their lives when they are deciding to give birth a life. In this context, strategies aimed at increasing the knowledge and powers of action of women and members of their communities by supporting them in the formulation of their birth plans, and finally to reduce the first delay, would be a necessity.

## Data Availability

The datasets used and analyzed during this study are available from the corresponding author upon reasonable request.

## Abbreviations

PBF: Performance-based financing
DRC: Democratic Republic of the Congo
HGR: General reference hospital
CS: Health centers
CSR: Reference health centers
SDGs: Sustainable development goals
